# Noncompliance with Therapeutic Guidelines for Chronic Hepatitis B Patients in Minas Gerais, Brazil

**DOI:** 10.3390/idr14060094

**Published:** 2022-11-28

**Authors:** Cristiane Faria Oliveira Scarponi, Marco Antônio Ferreira Pedrosa, Marcos Paulo Gomes Mol, Dirceu Bartolomeu Greco

**Affiliations:** 1 Research and Development Board, Ezequiel Dias Foundation, Belo Horizonte CEP 31510-010, MG, Brazil; 2 GeOpem Engenharia e Tecnologia Limitada, Rua Manoel Inácio Bezerra 192, Brejo Santo CEP 63260-000, CE, Brazil; 3 Faculty of Medicine, Federal University of Minas Gerais, Av. Prof. Alfredo Balena 190, Santa Efigênia, Belo Horizonte CEP 30130-100, MG, Brazil

**Keywords:** chronic hepatitis B, therapeutic guidelines, HBV management, antiviral treatment, public health, Brazil

## Abstract

Standardized treatment regimens for chronic hepatitis B (CHB) are crucial in suppressing viral replication, disease progression and the development of complications. However, information on routine compliance with such therapeutic recommendations in medical practice is rare. Aim: To evaluate the application of Brazilian therapeutic guidelines for CHB within the scope of the Unified Health System in Minas Gerais state. Four key recommendations from the national guidelines were compared with data from treated patients: (i) eligibility to start treatment; (ii) type of treatment applied; (iii) rescue antiviral therapy; and (iv) monitoring of virological response. Most physicians (69.8%) declared to adopt these guidelines, while 10 of them were unaware. However, according to the criteria established by the guidelines, only 39.5% of treated patients should have been considered “truly” eligible to start treatment and only 67.6% of these underwent the recommended pharmacological treatment. The virological response was laboratory monitored in just over a third of patients. Rescue therapy was adequately supplanted in 41.2% of patients previously treated with lamivudine. There was low compliance with national guidelines by public service physicians in Brazil, highlighting the need to raise awareness of the importance of its adherence to expand the control of CHB. Thus, increasing the adherence of health professionals to this tool is a current challenge for health institutions and managers.

## 1. Introduction

Chronic hepatitis B (CHB) remains a major public health challenge due to its high morbidity and mortality rates. In 2017, a global report on viral hepatitis indicated that most people do not have access to testing and/or treatments [1,2]. Scientific evidence suggests that antiviral therapy applied at an opportune time to patients who need to start treatment can delay the progression of liver disease, preventing 15–25% of premature deaths from cirrhosis, hepatic decompensation and hepatocellular carcinoma [3,4,5,6].

Several clinical and therapeutic guidelines have been published to assist health professionals in proper CHB management. Careful patient’s selection for treatment indication is recommended, particularly for those with a persistent serum elevation of HBV-DNA, increased serum concentrations of alanine aminotransferase (ALT) and/or evidence of advanced liver disease. In eligible patients for antiviral therapy, treatment should first be initiated with one of the recommended drugs (interferon, entecavir or tenofovir) and the therapeutic response effectiveness should be monitored regularly through the measurements of HBV-DNA, alanine aminotransferase and serological tests [7,8,9].

At the beginning of the 21st century, more than two million people chronically infected with HBV resided in Brazil [10]. Thus in 2002, the Ministry of Health standardized the “Clinical Protocol and Therapeutic Guidelines for Chronic viral Hepatitis B and Coinfections” (CPTG-CHB), aiming at guaranteeing the integrality of treatment and the prescription of safe and effective medications [11]. Later updates introduced new drugs with a sequential treatment approach, preserving future alternatives for possible viral resistance and therapeutic failure [12]. The national therapeutic guidelines for hepatitis B help physicians to prescribe drugs according to each clinical and laboratory situation, seeking the rational use of the therapeutic arsenal and the best cost-effectiveness [11,12]. The Brazilian therapeutic guidelines were based on the best published scientific evidence and agreed with the main international consensuses for the management of patients with CHB [7,8,9,12].

Information on routine compliance with such therapeutic recommendations in medical practice is rare. A large study conducted in the United States showed that adherence to AASLD guidelines for the management of CHB is very low, particularly regarding criteria for initiating treatment and monitoring the serologic response [6]. Other studies have shown some reluctance and/or lack of knowledge of the current guidelines for CHB therapeutic management, as evidenced by the poor adherence to this tool by physicians from different countries [13,14,15,16,17]. The present study examined the application of treatment guidelines in daily practice, using CPTG-CHB (v.2011) as a reference standard [12]. It assessed four key recommendations compared with data from patients who received treatment from the Unified Health System (SUS) in Minas Gerais, Brazil.

## 2. Methods

### 2.1. Population and Design Study

This is a complement analysis of a cross-sectional study based on secondary data (clinical, laboratory and therapeutic information) of CHB patients’ records sent to the Central Laboratory of Public Health of Minas Gerais, from May 2012 to September 2013 [18]. Minas Gerais is the second most populous state (with approximately 20 million inhabitants), located in the Southeast region (corresponding to 36.6% of confirmed hepatitis B cases in Brazil) [12]. Criteria for inclusion: chronic HBV-infection patients already experienced treatment, regardless of sex or age, and living in any city in the state. The exclusion criteria: unavailability of this information. The sample size (*n* = 151) was calculated with a precision of 5%, a confidence level of 97%, and an expected frequency of 5% for a population of 1,292,432 CHB individuals (report of hepatitis B and C seroprevalence in Minas Gerais Survey, 2009, unpublished data), and a 20% loss rate using StatCalc (Epi Info^TM^ v.7.2.1.0, CDC, Atlanta, GA, USA).

Data from each patient (epidemiological, clinical and laboratory information) were included in a digital spreadsheet, preserving anonymity. The form consisted of six main sections: (i) patient demographic information, such as sex, age and city; (ii) clinical profile and biochemical, serological, molecular and histochemical test results; (iii) justification for the HBV-DNA quantification test, either to evaluate treatment indication or laboratory monitoring; (iv) adherence to the national therapeutic guidelines; (v) antiviral therapy applied; and (vi) viral resistance suggestive scenarios.

### 2.2. Statistical Analysis

Descriptive results were expressed as absolute or relative frequency and median with interquartile range (IQR 25% and 75%), using the Epi Info^TM^ software, v.7.2.1.0 (CDC, Atlanta, GA, USA). QGIS software v.3.10 (QGIS Development Team) was used to represent the spatial distribution of treated CHB patients. For analysis, each patient was allocated according to their data in one of the four flowcharts or special situations described in the CPTG-CHB [19]. The clinical–laboratory profiles of the treated patients were compared with the recommended criteria for therapeutic conduct in four main points: (i) eligibility for treatment; (ii) type of treatment; (iii) medication used for antiviral therapy rescue; and (iv) laboratory monitoring, during and after treatment. The STROBE guidelines were used to report this study [20].

### 2.3. Ethical Considerations

This study was approved by the Fundação Ezequiel Dias Research Ethics Committee (approval number: 4,434,487) and conducted in accordance with the National Health Council of Brazil (Resolution 466/12) and the Helsinki Declaration of 1975. It was part of the “Laboratory surveillance applied to disease control and management of HBV, HCV and/or HIV infected patients among SUS users in Minas Gerais” project.

## 3. Results

**Figure 1:**
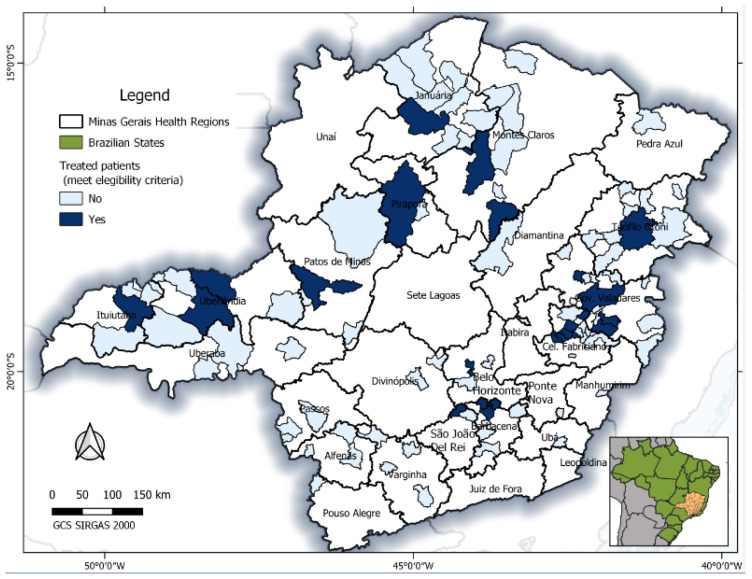
Geospatial location of treated CHB patients by health regions in the Minas Gerais state (Brazil), according to the eligibility criteria defined in the “Clinical Protocol and Therapeutic Guidelines for the Treatment of Chronic B Viral Hepatitis and Coinfections”, from May 2012 to September 2013, using QGIS software.

**Figure 2:**
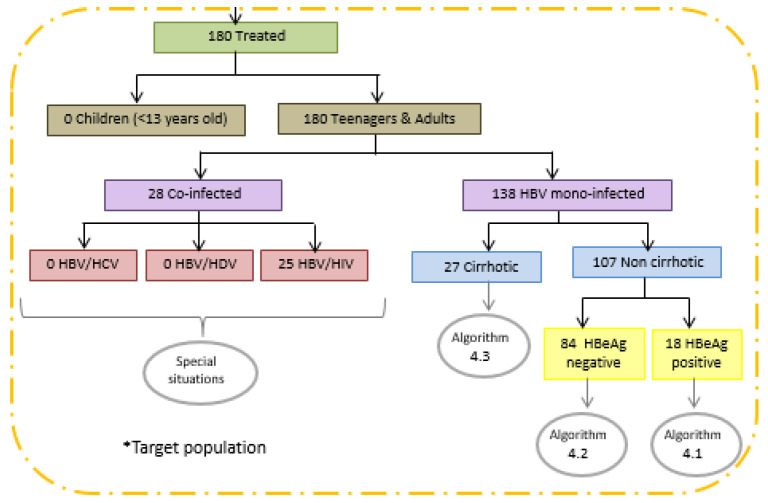
Flowchart of patients with HBC undergoing antiviral therapy, according to the eligibility criteria of the “Clinical Protocol and Therapeutic Guidelines for the Treatment of Chronic Viral Hepatitis B and Coinfections” (version 2011), Brazil. Legend: CHB, Chronic Hepatitis B; HBV, Hepatitis B virus; HBeAg, Hepatitis B virus “e” antigen; HCV, Hepatitis C virus; HDV, Hepatitis Delta virus; and HIV, Human Immunodeficiency virus. The number of individuals differs from the total due to a lack of data.

This study included 180 patients’ records with evidence of submission to antiviral treatment against HBV, residing in 58 (9.9%) cities of Minas Gerais, Brazil (Figure 1). Most of the treated CHB patients (140; 77.8%) were male, with a median age of 44 years (IIQ: 35.50–52.00), ranging from 18 to 84 years. The clinical–laboratory situation was predominantly non-cirrhotic (139; 83.2%), and normal ALT (111; 65.3%). Liver biopsy was performed in 32 (19.3%) treated patients, although the results were not available to all; some of them had moderate (≥A2) necroinflammatory activity (18; 69.2%), significant fibrosis (≥F2) and cirrhosis (18; 75.0%). Overall, 138 (83.3%) of them were HBV monoinfected; 136 patients (75.6%) performed HBV-DNA quantification, of which 117 (86.0%) had detectable levels and a 3.18 Log UI/mL (IIQ: 1.30–4.77) median viral load. Serology revealed a predominance of negative HBeAg (137; 80.6%) and positive anti-HBe (120; 75.5%) markers (Figure 2).

**Figure 3:**
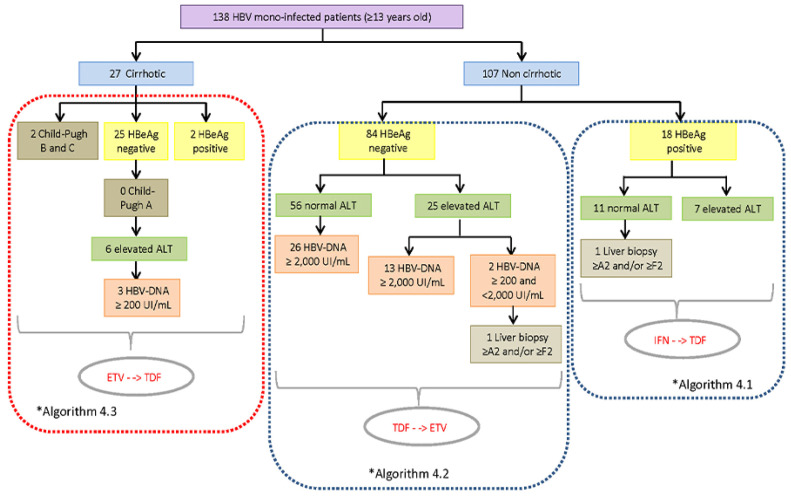
Distribution of treated CHB patients over 13 years, monoinfected and non-cirrhotic, eligible for antiviral therapy according to the algorithms “Clinical Protocol and Therapeutic Guidelines for the Treatment of Chronic Viral Hepatitis B and Coinfections” (version 2011) and evaluated from May 2012 to September 2013 at the Central Laboratory of Public Health of Minas Gerais (Brazil). Legend: ALT, Alanine aminotransferase; DNA, deoxyribonucleic acid; ETV, entecavir; HBV, Hepatitis B virus; HBeAg, Hepatitis B virus “e” antigen; IFN, Interferon; and TDF, Tenofovir. The number of individuals differs from the total due to a lack of data.

**Figure 4:**
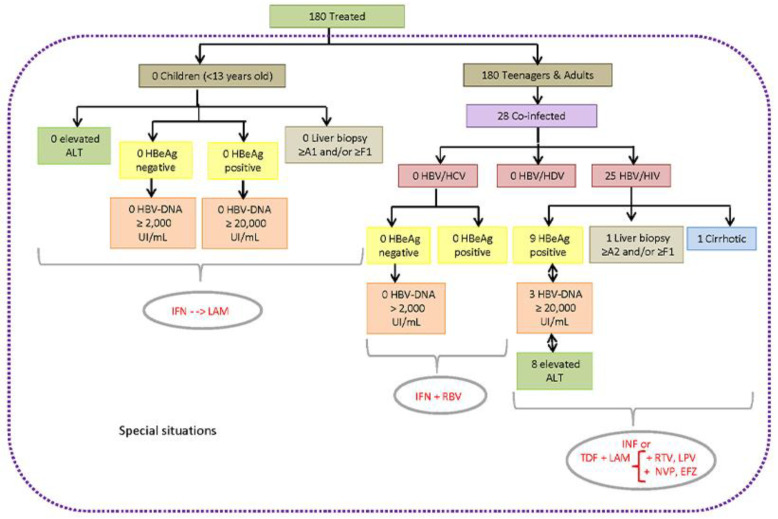
Distribution of treated CHB patients, classified as a special situation, eligible for antiviral therapy, according to the algorithms “Clinical Protocol and Therapeutic Guidelines for the Treatment of Chronic Viral Hepatitis B and Coinfections” (version 2011) and evaluated from May 2012 to September 2013 at the Central Laboratory of Public Health of Minas Gerais (Brazil). Legend: ALT, Alanine aminotransferase; DNA, Deoxyribonucleic acid; EFZ, Efanvirez; ETV, Entecavir; HBV, Hepatitis B virus; HBeAg, Hepatitis B virus “e” antigen; HCV, Hepatitis C virus; HDV, Hepatitis Delta virus; HIV, Human Immunodeficiency virus; IFN, Interferon; LAM, Lamivudine; LPV, Lopinavir, RBV, Ribavirin; RTV, Ritonavir; and TDF, Tenofovir. The number of individuals differs from the total due to a lack of data.

Focusing on the application of the CPTG-CHB guidelines, in 111 forms (91.7%) there was a positive sign of adoption by most physicians (44/63; 69.8%) responsible for patients’ therapeutic conduct. On the other hand, 10 of them (8.3%) were unaware of their existence or did not adhere to this tool. This adherence rate was not confirmed after comparisons between the clinical–laboratory data of treated patients and the therapeutic recommendations established in the CPTG-CHB. Of the 180 treated patients, only 71 (39.4%) met the criteria established for antiviral therapy indication; therefore, they were considered “truly” eligible according to this guideline (Table 1). The distribution of treatment-eligible CHB patients, according to the algorithms and special situations recommended in the CPTG-CHB, are shown in Figure 3 and Figure 4. In the 109 remaining patients (72.7%), the hepatic markers described in the forms did not justify the prompt introduction of antiviral therapy.

Considering the type of treatment instituted for the 159 CHB patients, there was a predominance of antiviral monotherapy in 143 patients initially. The medical preference for the first drug to be administered was tenofovir (67; 42.1%), followed by entecavir (45; 28.3%), lamivudine (17; 10.7%), interferon (10; 6.3%) and adefovir (4; 2.5%). Another 28 CHB patients were using combined therapies due to the presence of viral coinfections, mainly involving HBV/HIV (25; 89.3%). Surprisingly, a triple HBV/HCV/HIV infection was reported in two male patients (7.1%), both negative for HBeAg, undetectable HBV-DNA load and <2000 IU/mL for HCV-RNA (genotype 1B and 1A), undergoing treatment (one with tenofovir associated with lamivudine in combined therapy and the other, lamivudine monotherapy); one presenting normal ALT and the other, altered ALT.

**Figure 5:**
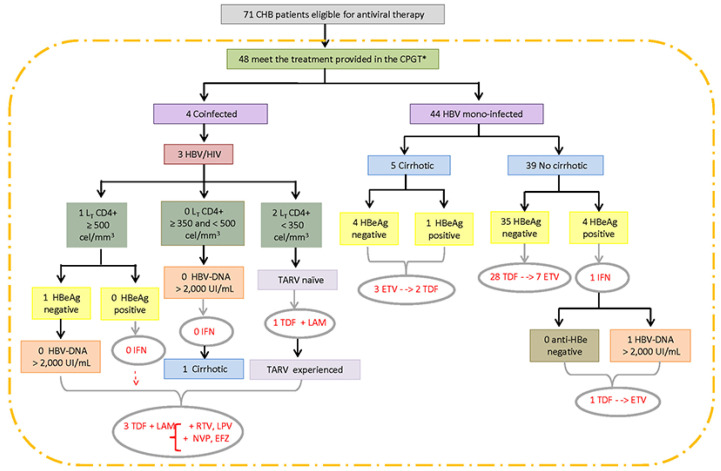
Flowchart of the disposition of treated CHB patients according to the therapeutic medication provided in the “Clinical Protocol and Therapeutic Guidelines for the Treatment of Chronic Viral Hepatitis B and Coinfections” (version 2011), Brazil. Legend: DNA, Deoxyribonucleic acid; EFZ, Efanvirez; ETV, Entecavir; HBV, Hepatitis B virus; HBeAg, Hepatitis B virus “e” antigen; HIV, Human Immunodeficiency virus; LAM, Lamivudine; LPV, Lopinavir, Lt CD4+, T-lymphocytes subsets CD4+; NVP, Nevirapine; RTV, Ritonavir; TARV, antiretroviral therapy; and TDF, Tenofovir. The number of individuals differs from the total due to a lack of data.

Laboratory monitoring (ALT and HBV-DNA levels) to verify the effectiveness of the therapeutic response was described in 41.1% of treated CHB patients (74/180) with the following regularity: every four months for one patient, semiannually for 49 patients, and annually for 24 patients. When restricting this analysis to “truly” eligible patients who complied with the recommended pharmacological indication, this monitoring was carried out in only half of them (24 treated patients). The suspicion of pre-core HBV mutant occurrence was reported in 17 patients; in addition to medication failure or change during treatment and lamivudine resistance in 10 patients. Among the 71 CHB patients considered eligible for the study, only 48 (67.7%) benefited from the recommended pharmacological treatment standard reference (Figure 5).

The patient therapeutic rescue, considering the concepts of therapy and rational sequential use of drugs provided for CPTG-CHB, is shown in Table 2. Regarding experimented lamivudine patients, rescue therapy was conveniently replaced in 41.2% (7/17) of the first recommended drug options tenofovir (4) and lamivudine-associated tenofovir combination (1), and the second drug option entecavir (1). In addition, the patient was treated with adefovir, which was not recommended in this case, according to the CPTG-CHB. Interferon-alpha was initially administered to 10 CHB patients; there was a suspicion of a pre-core HBV mutant posteriorly in four of them which were rescued with the first drug option foreseen for this situation, tenofovir, and in another patient, using the second option, entecavir. Among the patients previously submitted to antiviral therapy with another nucleotide analog(s), no exchange record was found when the first medication was tenofovir (67), entecavir (45) or adefovir (4) (Table 2).

## 4. Discussion

Treatment for CHB patient indication is never a simple decision for most physicians in the routine of health services, requiring special attention to disease stages and the constant updating of pharmacological options. Several therapeutic guidelines seek to help identify patients who are more likely to benefit from antiviral therapy. In addition, they guide physicians in the selection of safe and effective antiviral drugs [7,8,9,18,19,21]. However, this study shows that not all Brazilian physicians in the public health system, especially those responsible for patients with HBC undergoing treatment (8.26%), know or adopt the CPTG guidelines. A similar situation was observed in an American study, in which a significant number of physicians (62%) did not know the CHB treatment proposed guidelines [14]. Assessments on familiarity with therapeutic guidelines for the management of Hepatitis B and their applicability in medical practice are scarce, which underscores the importance of this comprehensive study.

Furthermore, the results highlight the enormous difficulty presented by more than two-thirds of physicians to adopt therapeutic guidelines in routine practice, even among those who expressed following this tool, leading to less than expected assistance. An American study reported that only one third of patients with CHB, seen by primary care physicians, were adequately evaluated for treatment indication, in addition to receiving older medication such as lamivudine [15]. CHB management decisions are not always based on scientific evidence. The routine application of these guidelines was also lower among Chinese and Spanish physicians, in which only half of them followed the CHB treatment criteria described [22,23]. These findings agree with several studies suggesting that, in real life, there is a lack of awareness and insufficient application of the current management guidelines for CHB patients among health professionals at different levels of care [13,14,15,16,17,24,25].

The present study also reveals which parameters were least adhered to by Brazilian physicians in the public system, based on the analysis of four therapeutic conduct main points, according to CPTG-CHB [19]. A substantial number of patients were indicated for treatment through uncertain criteria. The data showed a lack of criteria for antiviral therapy indication and the adoption of personal preferences when choosing the first scheme. In addition to a poor laboratory monitoring during and after drug introduction. It is emphasized that the CPTG-CHB contemplates all tests and pharmacological options available by the Brazilian Public Health System, with aim of providing free assistance and comprehensive treatment, in response to different patients’ access to health services and medium and high complexity procedures [19]. Greater dissemination of this tool and targeted medical education are necessary to address CHB knowledge gaps, which would result in a more assertive therapeutic approach and, therefore, better clinical-virological outcomes for patients.

One of the crucial issues in CHB management is the careful selection of eligible candidates for antiviral treatment. The clinical–laboratory information indicated that less than half of the treated patients met the CPTG-CHB eligibility criteria. In contrast, a significant proportion of patients undergoing treatment (72.7%) did not meet some of the criteria necessary to justify the immediate introduction of HBV therapy. This proportion is much higher than reported in a study conducted with 527 patients from the North and Northeast Regions of Brazil, in which 26% had no indications based on the same guidelines [25]. In another study carried out in the USA, these values were even more expressive compared to 11.64% of patients who demonstrated a lack of clarity in the eligibility requirements according to AASLD recommendations [26,27]. These findings reinforce the argument that physicians familiar with the guidelines are significantly more likely to identify the most suitable candidates for therapy [28].

Regarding the type of therapy, it is important to know which and how these choices are determined, especially as first-line treatment (or therapeutic regimen). In this study, the initial therapeutic regimen was predominantly monotherapy for 89.9% of treated CHB patients, with preference (93.0%) for nucleotide (s) analogs; the percentage was higher than the 68.2% of prescriptions reported in a similar study in other regions of Brazil [25]. The reasons for choosing tenofovir and entecavir as the preferred drugs for the first therapeutic regimen (70.4%) are probably due to both having a potent antiviral effect and low incidence of resistance [9,19,29]. This high percentage may also be due to the higher proportion of negative HBeAg patients, for whom the first recommended therapeutic option is tenofovir, and in case of contraindication, should be indicated entecavir [19]. These findings differ from those of a previous study that evaluated CHB treatment preferences among Asian physicians, in which lamivudine or interferon monotherapy was the first-line therapy [22]. It should be noted that contraindications to the recommended drugs were not evaluated in this study.

Data demonstrate that two-thirds of the “truly” eligible patients received the CPTG-CHB recommended therapies, and for the other patients, the choice of medications was according to the physician’s personal preference. A similar therapeutic approach was observed for patients who would be considered “ineligible” and in which the pharmacological prioritization is not clear. Lamivudine monotherapy (11 patients) should not be the first pharmacological option because of its low genetic barrier and, therefore, high resistance potential. A plausible explanation is that these patients were treated before the updated version of the guidelines. It is expected that the treatment of these patients will be preferably replaced by tenofovir, due to the possibility of cross-resistance to entecavir. Situations contradict the provisions of the guidelines for the rational use of the therapeutic arsenal, which in theory could compromise the sustainability of prolonged care in the public health system aiming to guarantee universal access to comprehensive HBV treatment [17]. The findings differ from those of a European Union study in which 48% of HBeAg-positive patients were not treated according to EASL recommendations, but the choice was based mainly on therapeutic efficacy and low cost [16]. Currently, World Health Organization guidelines recommend the preferential use of oral drugs (tenofovir or entecavir) for continuous first- and second-line treatment. Combined with the monitoring of viral response at least every 3 months for the first year. A therapeutic regimen indicated for patients eligible for treatment is much more restricted than the guidelines in force in Brazil [30].

The patients’ laboratory monitoring, during and after therapy, aims to assess the effectiveness of the established therapeutic scheme and the expected clinical and virological outcomes, important issues for which there is little information about current practices [17,25]. However, the tests required to monitor treatment were not performed during the evaluation period for many patients. This situation may reflect a lack of awareness or familiarity with the CPTG-CHB. In addition, in regions where health assistance is present and accessible, there are specific population groups that still face obstacles to comprehensive care, regardless of the provision of health services. These findings corroborate national data that point to the underutilization of health services [13]. Other studies have shown low adherence to the CHB treatment guidelines among physicians in the USA, especially regarding therapeutic response verification to better guide therapy decisions, using markers such as HBV quantification and alanine aminotransferase levels [24].

Rescue therapy was conveniently administered to 41.18% of patients previously treated with lamivudine. The first pharmacological options were substituted following the concept of sequential therapy in patients with antiviral therapy failure or resistance (tenofovir, lamivudine combined with tenofovir) and a second drug option, entecavir. There was a report of one adefovir-rescued patient, a drug, not CPTG-CHB recommended [19].

This study has some limitations. The factors contributing to the lack of CPTG-CHB knowledge for some physicians, despite being an application tool at the national level, could not be evaluated because of the retrospective nature of the study. Professionals who were aware of this tool also had very few justifications for not adhering to the recommended therapeutic guidelines. It should be noted that contraindications to the recommended drugs were not evaluated in this study. However, regarding the sample bias, it would not significantly affect the direction and strength of the results obtained, in view of the considerable number of physicians and municipalities involved in the state. Other limitations are related to incomplete records which made it difficult to assess certain aspects of medical care, such as a low diagnosis rate for certain coinfections (HCV, HDV and HIV), which would determine specific therapy and a more effective outcome for the patient, in addition to laboratory monitoring data after the introduction of antiviral therapy.

However, this study revealed important information about the clinical–demographic characteristics of treated CHB patients in the public health system, as well as the therapeutic standards that are routinely applied in Minas Gerais. Fundamental findings to fill the knowledge gaps of health managers and assist in planning interventions that lead to the improvement of this important tool, such as data on antiviral therapy use and the application of the guidelines for CHB management in different economic and geographical contexts are limited. The findings could be extended sparingly to other Brazilian regions, given the great diversity of patients with HBC and specialist doctors, in addition to covering health services of the most diverse complexities in a considerable number of municipalities.

Furthermore, as described in the methods section, a preview paper [18] addressed patients not yet undergoing any treatment for HBV, while this paper aimed at assessing whether the therapeutic approach applied to treated patients is consistent with the management recommendations officially established in Brazil. Both papers accessed secondary data available from records sent to the Central Laboratory of Public Health of Minas Gerais.

Adherence to the four main points of CHB therapeutic guidelines was found to be low by public service physicians in Minas Gerais, Brazil. This is a situation that urgently needs to be discussed and reviewed, especially considering that in the period from 2000 to 2021, 264,640 cases of hepatitis B were reported in Brazil, reaching 17,540 deaths related to this disease. Of these, 53.4% had hepatitis B as the underlying cause, mostly in the Southeast region of the country (40.6%). The distribution of confirmed cases of HBV shows that almost half of the total (48.8%) was concentrated in young individuals aged between 25 and 44 years [31].

Therefore, it becomes evident that there is a need for this tool to be more publicized, aiming to reach health professionals at all levels of health care in order to improve medical conduct and contribute to timely CHB control. Thus, increasing health professionals’ adherence to this tool is currently a challenge for health institutions and managers.

## Figures and Tables

**Table 1:** Profile of treated CHB patients, according to the algorithms “Clinical Protocol and Therapeutic Guidelines for the Treatment of Chronic Viral Hepatitis B and Coinfections” (version 2011) and evaluated from May 2012 to September 2013 at Central Laboratory of Public Health of Minas Gerais (Brazil).

Treatment Criteria (CPTG-CHB)	Clinical-Laboratory Profile of Patients	Number of Treated Patient’s	Number of Patients Who Meet Eligibility Criteria	% Conformity Eligibility Patient Selection	Therapeutic Options	Number of Eligible Patients Who Meet the Recommended Therapeutic Guidelines	% Conformity pharmacological Selection	% Overall of Non-Conformity
Algorithm 4.1	HBeAg positive, non-cirrhotic	18	8	44.4	IFN, TDF	4	50.0	22.2
Algorithm 4.2	HBeAg negative, non-cirrhotic	84	40	47.6	TDF, ETV	35	87.6	41.7
Algorithm 4.3	Cirrhotic with HBeAg positive or negative	27	6	22.2	ETV, TDF	5	83.3	18.5
Special situation	HBV-HIV Coinfection	25	16	64.0	IFN + RBV	3	18.7	12.0

Legend: CPTG-CHB, Clinical Protocol and Therapeutic Guidelines for the Treatment of Chronic Viral Hepatitis B; ETV, entecavir; HBV, Hepatitis B virus; HBeAg, Hepatitis B virus “e” antigen; IFN, Interferon; RBV, Ribavirin; and TDF, Tenofovir. The number of individuals differs from the total due to a lack of data.

**Table 2:** Distribution to rescue patients with CHB antiviral therapy applied, according to the algorithms “Clinical Protocol and Therapeutic Guidelines for the Treatment of Chronic Viral Hepatitis B and Co-infections” (version 2011) and evaluated from May, 2012 to September, 2013 at Central Laboratory of Public Health of Minas Gerais (Brazil).

Therapeutic in Use	First Redemption Option		Second Redemption Option	
17 LAM	1 LAM + TDF **	4 TDF	1 ETV	
1 LAM + ADF	0 LAM + TDF	0 TDF	0 ADV + ETV	0 ETV
4 ADF	0 LAM * + TDF **	0 TDF	0 ADV + ETV	0 ETV
10 INFα	4 TDF		0 ETV	
45 ETV	0 ETV + TDF	0 TDF	0 ADV + ETV	
67 TDF	Until time has no report of HBV resistant to TDF			

Legend: LAM, Lamivudine; ADF, Adefovir; TDF, Tenofovir; ETV, Entecavir; IFN, Interferon; HBV, Hepatitis B virus. * If you have not used lamivudine or resistence to it. ** Intolerance or contraindication to TDF: indicate rescue with LAM + ADF. Number of individuals differ from the total due to a lack of data.

## Data Availability

Not applicable.
